# The Negative Effect of Mean Perfusion Pressure on the Development of Acute Kidney Injury after Transcatheter Aortic Valve Implantation

**DOI:** 10.21470/1678-9741-2018-0137

**Published:** 2018

**Authors:** Ilker Gül, Levent Cerit, Bihter Senturk, Mustafa Zungur, Mustafa Beyazıt Alkan, Hatice Kemal, Zeynep Cerit, Belma Yaman, Songul Usalp, Hamza Duygu

**Affiliations:** 1 Near East University, Faculty of Medicine, Department of Cardiology, Nicosia, Cyprus.; 2 Dokuz Eylül University, Faculty of Medicine, Department of Cardiology, Izmir, Turkey.; 3 Kent Hospital, Cardiology Clinic, Izmir, Turkey.; 4 Kas State Hospital, Antalya, Turkey.; 5 Near East University, Faculty of Medicine, Department of Pediatric Cardiology, Nicosia, Cyprus.

**Keywords:** Transcatheter Aortic Valve Replacement, Kidney, Acute Kidney Injury, Perfusion

## Abstract

**Objective:**

To evaluate the predictive value of mean perfusion pressure (mPP) in the development of acute kidney injury (AKIN) after transcatheter aortic valve implantation (TAVI).

**Methods:**

One hundred and forty seven consecutive patients with aortic stenosis (AS) were evaluated for this study and 133 of them were included. Mean arterial pressure (mAP) and central venous pressure (CVP) were used to calculate mPP before TAVI procedure (mPP = mAP-CVP). The occurrence of AKIN was evaluated with AKIN classification according to the Valve Academic Research Consortium-2 recommendations. The patients were divided into two groups according to the receiver operating characteristic (ROC) analysis of their mPP levels (high-risk group and low-risk group).

**Results:**

The AKIN prevalence was 22.6% in this study population. Baseline serum creatinine level, glomerular filtration rate, amount of contrast medium, and the level of mPP were determined as predictive factors for the development of AKIN.

**Conclusion:**

The occurrence of AKIN is associated with increased morbidity and mortality rates in patients with TAVI. In addition to the amount of contrast medium and basal kidney functions, our study showed that lower mPP was strongly associated with development of AKIN after TAVI.

**Table:** 

Abbreviations, acronyms & symbols				
**AKIN**	**= Acute kidney injury**	** **	**GFR**	**= Glomerular filtration rate**
**AR**	**= Aortic regurgitation**		**HR-G**	**= High-risk group**
**AS**	**= Aortic stenosis**		**LR-G**	**= Low-risk group**
**AUC**	**= Area under the curve**		**mAP**	**= Mean arterial pressure**
**AVA**	**= Aortic valve area**		**mPP**	**= Mean perfusion pressure**
**CAD**	**= Coronary artery disease**		**NRF**	**= Normal renal functions**
**CHF**	**= Congestive heart failure**		**PCI**	**= Percutaneous coronary intervention**
**CI**	**= Confidence interval**		**RBC**	**= Red blood cell**
**CM**	**= Contrast mediums**		**ROC**	**= Receiver operating characteristic**
**COPD**	**= Chronic obstructive pulmonary disease**		**SAVR**	**= Surgical aortic valve replacement**
**CrCl**	**= Creatinine clearance**		**SBP**	**= Systolic blood pressure**
**CVP**	**= Central venous pressure**		**STS**	**= Society of Thoracic Surgeons**
**CT**	**= Computed tomography**		**TAVI**	**= Transcatheter aortic valve implantation**
**DBP**	**= Diastolic blood pressure**		**VARC-2**	**= Valve Academic Research Consortium-2**
**DM**	**= Diabetes mellitus**			

## INTRODUCTION

Aortic stenosis (AS) is one of the most common cardiac degenerative valvular diseases, with a prevalence of 3-5% in patients above 75 years of age^[1]^. Surgical aortic valve replacement (SAVR) is currently considered the gold standard treatment for severe symptomatic AS^[2]^. Transcatheter aortic valve implantation (TAVI) has emerged as an alternative to surgery for patients with severe symptomatic AS, particularly for those who were considered at intermediate to high risk for surgery^[3,4]^. Since its introduction in 2002, more than 200,000 patients have undergone TAVI globally.

Ageing, preexisting impaired kidney function, hemodynamic instability, congestive heart failure (CHF), diabetes mellitus (DM), anaemia, and the usage of great amount of contrast mediums (CM) are well-known risk factors for the development of acute kidney injury (AKIN) after TAVI^[5,6]^.

Although these risk factors have been well known, the predictive value of mean perfusion pressure (mPP) in the development of AKIN has not been investigated yet. Therefore, in this study, we aimed to evaluate the predictive value of mPP in the development of AKIN after TAVI.

## METHODS

In this study, 147 consecutive patients who had undergone TAVI procedure in our clinic between June 2013 and December 2015 were evaluated. One hundred and thirty three of them met the inclusion criteria and were included in this study. Patients who had invasive blood pressure monitorization, jugular venous catheter, and were hemodynamically stable were included in our study. Patients who had undergone renal replacement therapy, presented glomerular filtration rate (GFR) <30 ml/min/1.73 m^2^, had decompensated heart failure, received inotropic agents, had intra-aortic balloon pump, and received CM within the last 48 hours were excluded. Patients who had undergone additional procedures or received additional CM due to vascular complications, and had chronic pulmonary diseases or chronic liver diseases were also excluded from the study. Intravenous hydration therapy was started in patients with GFR <50 ml/min/1.73 m^2^ for 12 hours before the procedure and continued for 24-48 hours after TAVI. Patients' daily blood tests including creatinine were checked for three days before and three days after TAVI.

### Mean Perfusion Pressure

Blood pressure of every patient was monitored using invasive monitorization during the 12 hours before TAVI procedure. Also during this period, mean arterial pressure (mAP) calculated by monitors was entered in patient files. Central venous pressure (CVP) was monitored with catheters implanted by anesthesiologists in all patients before valve implantation. The mPP was calculated using the formula MPP = mAP-CVP.

### Acute Kidney Injury

The Valve Academic Research Consortium-2 (VARC-2) criteria were used to evaluate any complication occuring in TAVI patients^[7]^. VARC-2 recommends that the AKIN system should be used to diagnose AKIN. According to the AKIN system:

- AKIN Stage 1: Increase in serum creatinine of 150-199% (1.5-1.99 × increase compared with baseline) or increase of ≥0.3 mg/dL (≥26.4 mmol/L) or urine output <0.5 mL/kg/h for >6 h but <12 h;
- AKIN Stage 2: Increase in serum creatinine of 200-299% (2.0-2.99 × increase compared with baseline) or urine output <0.5 mL/kg/h for >12 h but <24 h;
- AKIN Stage 3: Increase in serum creatinine of ≥300% (>3 × increase compared with baseline) or serum creatinine of ≥4.0 mg/dL (≥354 mmol/L) with an acute increase of at least 0.5 mg/dL (44 mmol/L) or urine output <0.3 ml/kg/h for >24 h or anuria for >12 h.

### TAVI Procedure

Severe AS was diagnosed with echocardiographic methods. The situations of average aortic gradient > 40 mmHg, aortic valve area (AVA) <1 cm^2^, and valve area index (valve area/body surface area) <0.6 cm^2^ were considered to be severe AS^[8]^. The balloon-expandable Edwards Sapien XT valve (Edwards Lifesciences, Irvine, California, USA) was used for TAVI process. Vascular occlusion device (ProStar XL, Abbott Laboratories, North Chicago, Illinois, USA) was used in eligible patients in terms of femoral artery diameter and anatomy. The surgical cut-down method was applied in patients who were unsuitable for using iliac and femoral artery anatomy vascular closure device. Transoesophageal echocardiography and multislice computed tomography (CT) tests were done to determine the diameter of the aortic bioprosthesis implanted. In all patients, clopidogrel 75 mg and acetylsalicylic acid 100 mg were started before TAVI procedure. İohexol (Omnipaque, GE Healthcare), a nonionic low-osmolar monomeric CM, was used as the opaque material. The amount of CM was recorded during all TAVI procedure. Examinations such as CT and coronary angiography that required administration of CM, except TAVI procedures, were performed at least 72 hours before. Daily kidney function tests of all patients were monitored in our centre from admission to discharge. Creatinine levels were checked in the second week, and the first, third, sixth and twelfth month after TAVI procedure (COBAS Integra 400 plus, Roche Diagnostics).

The patients were prospectively followed during one year after TAVI. The informed consent form was obtained from each subject, and the study was conducted in accordance with the Helsinki Declaration. The study protocol was approved by the local ethics committee.

### Study Groups

Based on the receiver operating characteristic (ROC) analysis, patients who had lower mPP values (<72 mmHg), which was determined as the threshold value for AKIN development, were included in the high-risk group (HR-G), and those who had higher mPP values (≥72 mmHg) were included in the low-risk group (LR-G). Additionally, characteristics of patients with normal renal functions (NRF) and those who developed AKIN were evaluated.

### Statistical Analysis

Statistical analysis was performed using SPSS 17.0 software (SPSS, Chicago, Illinois, USA). The Kolmogorov-Smirnov test was used to assess the normality of distributions. Variables not normally distributed were expressed as medians (interquartile ranges). Normally distributed continuous variables were expressed as a mean ± standard deviation. The means for normally distributed continuous variables were compared by independent-samples t-tests. Skew-distributed continuous variables were compared using a Mann-Whitney U test. Pearsons x^2^ test and Fisher exact test were used to compare categorical variables. Univariate analyses were performed with the variables, such as age (years), left ventricular ejection fraction (%), mean aortic gradient (mmHg), Society of Thoracic Surgeons (STS) score (%), logistic EuroSCORE (%), mPP (mmHg), systolic blood pressure (SBP, mmHg), diastolic blood pressure (DBP, mmHg), DM (%), hypertension (%), coronary artery disease (CAD, %), creatinine (mg/dl), GFR (ml/dk/1.73 m^2^), amount of CM (ml), pre-TAVI haemoglobin levels (g/dl), and red blood cell transfusion after TAVI (%). The backward stepwise multivariate regression analysis was performed with the variables of mPP, left ventricular ejection fraction, baseline creatinine, GFR, red blood cell (RBC) transfusion, amount of CM, SBP, and DBP; the *P* values of those was found to be *P*<0.10, by univariate analyses. A ROC curve analysis was performed to identify the optimal cut-off point of mPP to predict AKIN in patients with severe AS. The area under the curve (AUC) values were calculated as a measure of test accuracy. A two-sided *P*<0.05 was considered significant within a 95% confidence interval (CI). Kaplan-Meier survival plots were constructed from the index procedure and up to one year after that and compared using the log-rank test. *P* values <0.05 were accepted as statistically significant.

## RESULTS

### Patients' General Characteristics

One hundred and thirty three patients (54.1% females; mean age of 78.1±7.5 years) were enrolled in this study. AKIN was recorded in 30 patients after TAVI (22.6%). Mean values of SBP, DBP, mAP, and mPP were lower in HR-G and AKIN groups. The mean value of CVP was higher in HR-G and AKIN groups. The average mean gradient of the aortic valve was 49.7±11.7 mmHg, the mean AVA was 0.65±0.11 cm^2^, and the mean value of left ventricular ejection fraction was 42 ±14.7%.

### Procedural Data

A balloon-expandable Edwards SAPIEN XT valve was implanted via transfemoral access in all patients. The mean radiation time was 7.6±3.1 min. Mean CM and duration of procedure were 143.4±22.7 ml and 69.2±28.1 min, respectively. There weren't any significant difference between the two groups according to the numbers and durations of the rapid ventricular pacing.

### Mean Perfusion Pressure and Acute Kidney İnjury

The values of mPP were significantly lower in AKIN patients (65.9±9.5 *vs*. 76.3±7.4 *P*<0.001). Age, gender, DM, hypertension, chronic obstructive pulmonary disease (COPD), CAD, moderate to high grade aortic regurgitation (AR) after TAVI, risk scores for TAVI, haemoglobin, cardiac systolic functions, mean aortic gradient, kidney function tests, amount of CM, previous coronary bypass surgery, and percutaneous coronary intervention (PCI) rates were similar between the two groups. SBP, DBP, mAP, and mPP levels were significantly lower in HR-G group, but CVP level was higher in HR-G group (Table 1).

**Table 1:** Clinical, laboratory, echocardiographic, and angiographic characteristics of the study population

	HR-G (n=52)	LR-G (n=81)	*P* value
Age (years)	78.8±6.9	78.6±7.8	0.369
Female gender, n (%)	29 (55.8)	43 (53.1)	0.451
STS score (%)	13.3±4.3	14.6±6.7	0.472
Logistic EuroSCORE (%)	29.4 (15.9-38.2)	24.7 (14.8-35.3)	0.093
AKIN, n (%)	22 (42.3)	8 (9.9)	<0.001
Mortality, n (%)	5 (9.6)	3 (3.7)	0.153
Hemoglobin (g/dl)	12.3±1.6	12.4±1.8	0.860
RBC transfusion, n (%)	15 (28.8)	20 (24.7)	0.785
Left ventricle ejection fraction (%)	41.9±10.4	42.6±11.1	0.726
NT-pro BNP (pg/ml)	4188±1388	3811±1258	0.640
AVA (cm^2^)	0.65±0.19	0.64±0.21	0.506
Mean gradient (mmHg)	49.1±12.9	50.2±10.6	0.590
SBP (mmHg)	120.1±9.8	132.7±7.3	<0.001
DBP (mmHg)	57.4±8.1	69.6±9.4	<0.001
mAP (mmHg)	78.1±9.7	90.5±8.3	<0.001
CVP (mmHg)	13.2±2.9	10.9±2.5	<0.001
mPP (mmHg)	64.1±7.1	79.7±5.3	<0.001
Creatinine (mg/dl)	1.06±0.32	1.01±0.29	0.642
eGFR (ml/dk/1.73 m^2^)	60.15±14.6	62.69±16.7	0.348
Diuretic, n (%)	10 (19.2)	13 (16.0)	0.443
RAAS blocker, n (%)	24 (46.2)	40 (49.4)	0.214
Beta-blocker, n (%)	29 (55.7)	50 (61.7)	0.494
Amount of contrast (ml)	147 (115-245)	140.5 (120-212)	0.065
AR after TAVI (≥ grade II)	6 (11.5)	10 (12.3)	0.646
Number of rapid pacing	2.9±0.6	2.7±0.4	0.348
Rapid pacing duration (second)	41 (25-63)	40 (21-55)	0.642
Previous CABG, n (%)	10 (19.2)	13 (16.0)	0.443
Previous PCI, n (%)	9 (17.3)	19 (23.8)	0.254
Diabetes mellitus, n (%)	19 (36.5)	27 (33.3)	0.422
Hypertension, n (%)	21 (40.6)	34 (41.9)	0.908
Hypercholesterolemia, n (%)	20 (38.5)	40 (49.4)	0.145
COPD, n (%)	20 (38.5)	26 (32,1)	0.285
CAD, n (%)	25 (48.1)	40 (49.4)	0.512
Intensive care unit (days)	2 (1.3-3.2)	1.5 (1.1-2.1)	0.022
Hospital duration (days)	5 (3.5-6.2)	4 (3.3-5.8)	0.027

Values are number (%), mean ± standard deviation, or median [25^th^, 75^th^ percentiles].

AKIN=acute kidney injury; AR=aortic regurgitation; AVA=aortic valve area; BNP=b-type natriuretic peptide; CABG=coronary artery bypass graft; CAD=coronary artery disease; COPD=chronic obstructive pulmonary disease; CVP=central venous pressure; DBP=diastolic blood pressure; eGFR=estimated glomerular filtration rate; HR-G=High-risk group; LR-G=Low-risk group; mAP=mean arterial pressure; mPP=mean perfusion pressure; NT=N-terminal; PCI=percutaneous coronary intervention; RAAS=renin-angiotensin-aldosterone system; RBC=red blood cell; SBP=systolic blood pressure; STS=Society of Thoracic Surgeons; TAVI=transcatheter aortic valve implantation

### AKI and Risk Factors

There was no significant difference between AKIN and NRF groups in terms of CAD, DM, hypertension, and COPD. The AKIN group had longer hospitalisation duration (5.5 *vs*. 4 days, *P*=0.014). After TAVI procedure, 2 (2.2%) patients needed permanent pacemaker implantation due to atrioventricular conduction block. Transfusion rates were higher in the AKIN group (13 *vs*. 22, *P*=0.032). Patients in AKIN and NRF groups did not show a significant difference in terms of diuretic, renin-angiotensin-aldosterone blocker, and beta-blocker therapy (Table 1). The amount of CM was significantly higher in the AKIN group (148 *vs*. 138 ml, *P*=0.028). Eight patients died during the study period. Total mortality was higher in the AKIN group than in the NRF group (16.6% *vs*. 2.9%, *P*=0.015; Table 2).

**Table 2:** Baseline characteristics in patients with or without AKIN (NRF group).

	AKIN group (n=30)	NRF (n=103)	*P* value
Age (years)	78.2±7.3	77.6±8.1	0.699
Female gender, n (%)	14 (46.7)	58 (56.3)	0.234
STS score (%)	14.4±5.3	13.2±5.7	0.154
Logistic EuroSCORE (%)	27.5 (14.7-36.1)	26.5 (15.0-37.1)	0.742
Mortality, n (%)	5 (16.6)	3 (2.9)	0.015
Hemoglobin (g/dl)	11.1±1.7	11.3±1.8	0.716
RBC transfusion, n (%)	13 (43.3)	22 (21.4)	0.032
Left ventricle ejection fraction, %	41.9±10.4	42.6±11.1	0.245
NT-pro BNP (pg/ml)	4315±1492	3656±1168	0.524
AVA (cm^2^)	0.61±0.22	0.68±0.28	0.605
Mean gradient (mmHg)	51.5±13.8	49.2±10.8	0.355
SBP (mmHg)	121.9±10.6	129.5±9.7	<0.001
DBP (mmHg)	57.8±8.1	66.9±7.7	<0.001
mAP (mmHg)	79.5±7.8	87.5±6.9	<0.001
CVP (mmHg)	13.6±3.2	11.2±2.6	<0.001
mPP (mmHg)	65.9±9.5	76.3±7.4	<0.001
Creatinine (mg/dl)	1.17±0.36	0.99±0.26	0.003
eGFR (ml/dk/1.73 m^2^)	53.4±15.0	64.1±15.4	0.001
Diuretic, n (%)	8 (26.7)	15 (14.6)	0.548
RAAS blocker, n (%)	16 (53.3)	48 (46.6)	0.214
Beta-blocker, n (%)	18 (60.0)	51 (49.5)	0.406
Amount of contrast (ml)	148 (114-258)	138 (120-225)	0.028
AR after TAVI (≥ grade II), n (%)	5 (16.7)	11 (10.7)	0.646
Number of rapid ventricular pacing, n	3±0.8	2.7±0.4	0.442
Rapid pacing duration (second)	43 (26-67)	40.5 (22-53)	0.721
Previous CABG, n (%)	7 (23.3)	16 (15.5)	0.143
Previous PCI, n (%)	8 (26.7)	20 (19.4)	0.254
Diabetes mellitus, n (%)	10 (33.3)	36 (35.0)	0.527
Hypertension, n (%)	11 (36.7)	44 (42.7)	0.174
Hypercholesterolemia, n (%)	11 (36.7)	49 (47.6)	0.199
COPD, n (%)	12 (40.0)	34 (33.0)	0.309
CAD, n (%)	14 (46.7)	51 (49.5)	0.424
Intensive care unit (days)	2 (1.3-3.4)	1.5 (1.1-1.8)	0.001
Hospital duration (days)	5.5 (3.4-6.8)	4 (3.1-5.2)	0.014

Values are number (%), mean ± standard deviation, or median [25^th^, 75^th^ percentiles].

AKIN=acute kidney injury; AR=aortic regurgitation; AVA=aortic valve area; BNP=b-type natriuretic peptide; CABG=coronary artery bypass graft; CAD=coronary artery disease; COPD=chronic obstructive pulmonary disease; CVP=central venous pressure; DBP=diastolic blood pressure; eGFR=estimated glomerular filtration rate; HR-G=High-risk group; LR-G=Low-risk group; mAP=mean arterial pressure; mPP=mean perfusion pressure; NT=N-terminal; PCI=percutaneous coronary intervention; RAAS=renin-angiotensin-aldosterone system; RBC=red blood cell; SBP=systolic blood pressure; STS=Society of Thoracic Surgeons; TAVI=transcatheter aortic valve implantation

### AKIN Predictors and Survival

**Fig. 1:**
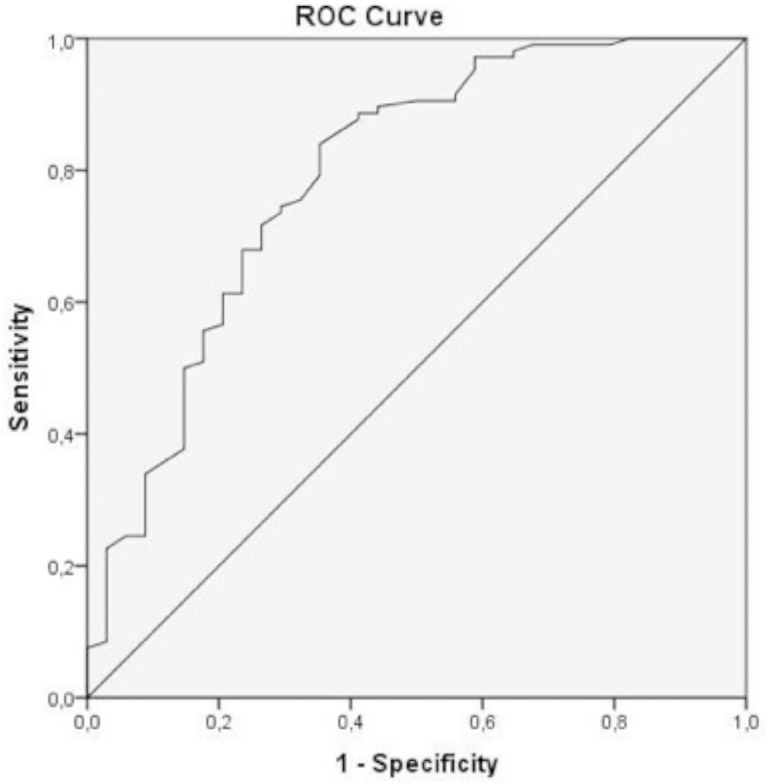
ROC curve of mean perfusion pressure. The mean perfusion pressure value which can predict the acute kidney injury development was determined as 72 mmHg in ROC analysis [AUC: 0.813 (95% CI; 0.721-0.905); sensitivity, 72%; specificity, 84%]. AUC = area under the curve; CI = confidence interval; ROC = receiver operating characteristics.

**Fig. 2:**
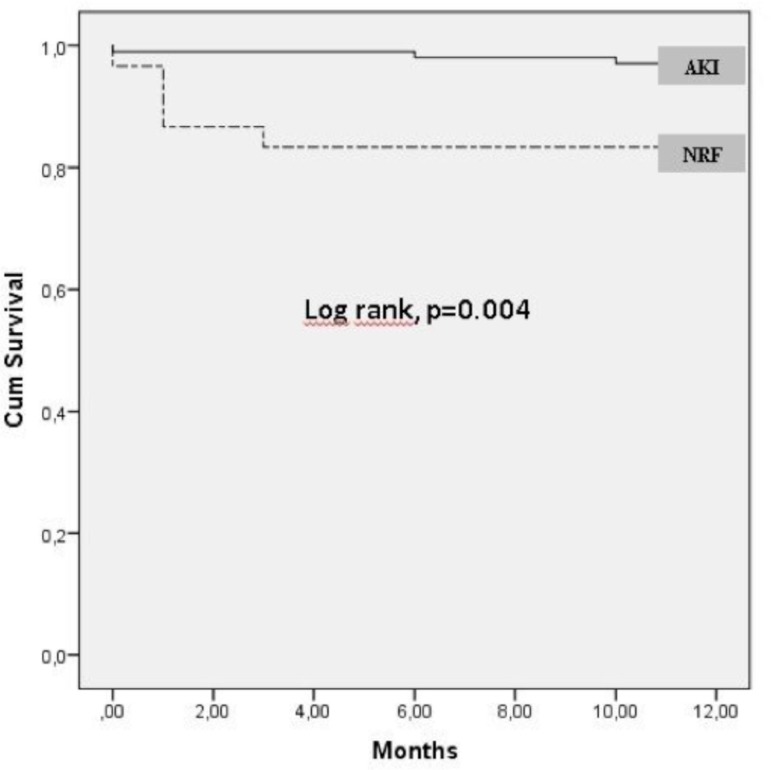
Kaplan-Meier curves for overall survival in AKIN and non-AKIN (NRF) groups. AKIN = acute kidney injury; NRF = normal renal functions.

In univariate analysis, mPP, left ventricle ejection fraction, baseline creatinine, GFR, red blood cell transfusion, amount of CM, SBP, and DBP were found to be significantly associated with AKIN *(P*<0.01 for all parameters). Thus, the multivariate regression analysis was performed with these variables; baseline creatinine, baseline GFR, amount of CM, and mPP were found to be significant predictors of AKIN (Table 3). The ROC analysis of the significant variables in multivariate regression analysis revealed that the cut-off value of mPP was 72 mmHg to predict the development of AKIN (AUC, 0.813; 95% CI, 0.721-0.905; sensitivity, 72%; specificity, 84%; Figure 1). Mortality rates were significantly higher in AKIN patients. Kaplan-Meier survival curves for patients with and without AKIN (NRF group) showed a significantly lower survival rate up to 1 year in the overall AKIN group (16.6% *vs*. 0.03% log-rank, *P*=0.02; Figure 2).

**Table 3:** Results of multivariate regression analysis for predictors of post-TAVI AKIN.

	Odds ratio	95% CI	*P* value
mPP (mmHg)	5.1	2.7-8.5	0.013
Amount of contrast (ml)	7.0	3.2-11.1	0.008
RBC transfusion, n	0.7	0.3-1.4	0.385
Baseline creatinine (mg/dl)	2.1	1.1-3.3	0.044
Baseline GFR (ml/min/1.73 m^2^)	2.6	1.4-4.1	0.032
SBP (mmHg)	0.5	0.2-1.1	0.782
DBP (mmHg)	0.6	0.4-1.1	0.582
Left ventricle ejection fraction (%)	1.3	0.7-2.0	0.248

AKIN=acute kidney injury; CI=confidence interval; DBP=diastolic blood pressure; GFR=glomerular filtration rate; mPP=mean perfusion pressure; RBC=red blood cell; SBP=systolic blood pressure; TAVI=transcatheter aortic valve implantation

## DISCUSSION

In our study, we found out that baseline creatinine, GFR, amount of CM, and lower mPP values were significantly associated with the development of AKIN in patients with TAVI.

Development of AKIN is strongly associated with increased major adverse cardiac events after TAVI procedure^[9-11]^. While 40% of the patients suffer from AKIN after SAVR, prevalence of AKIN ranges from 3.4% to 57% after TAVI procedure^[10,12,13]^. In accordance with previous studies, in our study 33 (21.2%) patients developed AKIN after TAVI. Excessive CM usage, hypotension, rapid ventricular pacing, balloon aortoplasty, valve implantation, and embolisation of aortic plaque are considered intra-operative risk factors for AKIN after TAVI^[12,14]^.

As it is well known, CM reduce oxygen availability to the renal medulla and cause renal ischemia^[15]^. Several studies demonstrated that CM/GFR and CM/creatinine clearance (CrCl) rates in invasive procedures are predictors of renal failure^[16-18]^. It can be useful to calculate basal GFR and CrCl before TAVI, to be used to calculate upper limits for CM. The mAP might be decreased below 50 mmHg during rapid ventricular pacing. These hypotensive stages can contribute to the development of AKIN. Therefore, number and duration of rapid ventricular pacing used for our patients during TAVI were recorded in our study. Although basal kidney function tests are important for impairment of renal function, other factors, such as mPP, amount of CM, and rapid ventricular pacing, should be kept in mind to predict renal impairment.

In addition to many factors affecting the development of AKIN, hemodynamic parameters of patients before an invasive procedure are also important indicators of potential renal complications. Renal hypoperfusion is the most important cause of AKIN after SAVR and TAVI^[11,19]^. Renal perfusion pressure is the most important predictor of renal blood flow. A normal renal perfusion pressure should be between 60 and 100 mmHg^[20]^. However, there isn't any non-invasive method that can directly measure it. Renal perfusion pressure can be estimated using mPP, mAP, and CVP levels. Especially, mPP calculated with mAP and CBP is shown to be an important indicator for the continuation of NRF^[21]^. A strong association has been demonstrated between mPP levels and GFR in various diseases^[22]^. Based on the study results, mPP values obtained with invasive monitorization in advanced AS patients were found to be a significant factor in predicting the development of AKIN. The predictive value of AKIN development was calculated as 72 mmHg using ROC analysis (Figure 1). The percentage of AKIN was much higher in the HR-G group (42.3% *vs*. 9.9%, *P*<0.001). The calculation of mPP is based on the difference between mAP and CVP. Since mAP values of the HR-G group were lower and CVP values were higher, mPP values in this group were low (Table 1). The value of mPP is more closely associated with renal perfusion pressure, compared to mAP and CVP^[21,22]^.

### Limitations of the Study

The main limitation of this study is the small number of patients included. Additional information can be obtained in longer follow-up periods. In our study, TAVI using balloon expandable prosthesis was performed, and it would be useful to conduct similar studies with a self-expandable prosthesis.

## CONCLUSION

These findings led to the conclusion that among the patients with similar renal functions, who received a similar amount of CM, those with lower mPP are at higher risk for AKIN development. A model was created with regression analysis to identify the factors affecting the development of AKIN in our study.

The mortality rate in patients who developed AKIN after TAVI ranges from 7.8% to 29%^[6,23]^. In our study, the one-year mortality rate was 16.7% in patients who developed AKIN and 2.9% in patients who did not. The presence of AKIN increased the mortality in a one-year period approximately by 5.5 times. Therefore, preventing the development of AKIN should be an important goal to minimise TAVI complications.

In our study, the amount of CM, basal kidney function tests, and lower mPP levels (mPP <72 mmHg) were strongly associated with the development of AKIN after TAVI. Further studies are needed to evaluate the association between mPP and AKIN in patients treated with TAVI.

**Table:** 

**Authors’ roles & responsibilities**	
IG	Analysis and interpretation of data; drafting the paper; revising the work; approval of the final version
LC	Analysis and interpretation of data; drafting the paper; revising the work; approval of the final version
BS	Conception and design of the work; acquisition of data; analysis and interpretation of data; drafting the paper; revising the work; approval of the final version
MZ	Conception and design of the work; acquisition of data; analysis and interpretation of data; drafting the paper; revising the work; approval of the final version
MBA	Conception and design of the work; acquisition of data; analysis and interpretation of data; drafting the paper; revising the work; approval of the final version
HK	Conception and design of the work; acquisition of data; analysis and interpretation of data; drafting the paper; revising the work; approval of the final version
ZC	Conception and design of the work; acquisition of data; analysis and interpretation of data; drafting the paper; revising the work; approval of the final version
BY	Conception and design of the work; acquisition of data; analysis and interpretation of data; drafting the paper; revising the work; approval of the final version
SU	Conception and design of the work; acquisition of data; analysis and interpretation of data; drafting the paper; revising the work; approval of the final version
HD	Conception and design of the work; acquisition of data; analysis and interpretation of data; drafting the paper; revising the work; approval of the final version
